# Recent Advances to Understand Morphology Stability of Organic Photovoltaics

**DOI:** 10.1007/s40820-016-0107-3

**Published:** 2016-10-04

**Authors:** Antonio Guerrero, Germà Garcia-Belmonte

**Affiliations:** Institute of Advanced Materials (INAM), Universitat Jaume I, 12006 Castelló, Spain

**Keywords:** Organic photovoltaics, Intrinsic degradation, Morphology, Thermal degradation, Interface

## Abstract

Organic photovoltaic devices are on the verge of commercialization with power conversion efficiencies exceeding 10 % in laboratory cells and above 8.5 % in modules. However, one of the main limitations hindering their mass scale production is the debatable inferior stability of organic photovoltaic devices in comparison to other technologies. Adequate donor/acceptor morphology of the active layer is required to provide carrier separation and transport to the electrodes. Unfortunately, the beneficial morphology for device performance is usually a kinetically frozen state which has not reached thermodynamic equilibrium. During the last 5 years, special efforts have been dedicated to isolate the effects related to morphology changes taking place within the active layer and compare to those affecting the interfaces with the external electrodes. The current review discusses some of the factors affecting the donor/acceptor morphology evolution as one of the major intrinsic degradation pathways. Special attention is paid to factors in the nano- and microscale domain. For example, phase segregation of the polymer and fullerene domains due to Ostwald ripening is a major factor in the microscale domain and is affected by the presence of additives, glass transition temperature of the polymers or use of crosslinkers in the active layer. Alternatively, the role of vertical segregation profile toward the external electrodes is key for device operation, being a clear case of nanoscale morphology evolution. For example, donor and acceptor molecules actually present at the external interfaces will determine the leakage current of the device, energy-level alignment, and interfacial recombination processes. Different techniques have been developed over the last few years to understand its relationship with the device efficiency. Of special interest are those techniques which enable in situ analysis being non-destructive as they can be used to study accelerated degradation experiments and some will be discussed here.

## Introduction

Organic photovoltaic technology are on the verge of commercialization with power conversion efficiencies (PCE) exceeding 10 % in laboratory cells [1] and above 8.5 % in modules [2]. A wide range of consumable applications may be reached, thanks to these PCE values and to the potentially low production costs and light weight. However, one of the main limitations hindering its mass scale production is the arguably low stability of organic photovoltaic. Stability requirements depend exclusively on the application where the organic photovoltaic (OPV) will be used. For example, requirements for futuristic applications like single-use/disposable electronics will be very mild with short degradation times in the range of months may be permissible. On the other hand, requirements for other applications like building integrated devices are highly demanding with a threshold of 10 % efficiency drop over a period of 20 years.

Significant efforts have been devoted to understand degradation processes of OPV and comprehensive reviews can be found in literatures [3–6]. In general, degradation mechanisms can be divided into extrinsic and intrinsic factors. External agents such as water [7], oxygen [8], light [9, 10], or heat [11] that may trigger accelerated degradation pathways constitute extrinsic degradation sources. If these external factors are controlled, degradation kinetics of the devices can be reduced. For example, encapsulation/lamination of devices is the most common practice to create a physical barrier for water and oxygen permeation into the device. Similarly, the use of UV filters dramatically reduces the photodegradation of the active layer [3]. Intrinsic degradation pathways include electrode diffusion, morphology evolution, or generation of charge transfer complexes between donor and acceptor molecules which act as source of photobleaching and recombination centers [12, 13].

Adequate donor/acceptor morphology of the active layer is required in the nanoscale to provide carrier separation and transport to the electrodes. Unfortunately, the beneficial morphology for device performance is usually a kinetically frozen state which has not reached thermodynamic equilibrium. For this reason, donor/acceptor morphology evolution during device operation is one of the major intrinsic degradation pathways and this topic will be discussed in detail in the present manuscript. Strictly speaking, degradation pathways due to light soaking and high temperature cannot be regarded as intrinsic processes. However, devices under operation conditions will always be under light and will suffer some degree of heating. Then, it is clear that these two factors can also be regarded as intrinsic factors. In general, light and heat induce morphology evolution of the active layer [14], interlayer and electrode diffusion [15], and electrode interaction with the organic materials [16].

The present manuscript aims at providing the latest developments to understand morphological degradation pathways present in most organic photovoltaics (OPVs). Recent advanced techniques to understand morphology evolution are presented. In addition, their use in some recent degradation studies is discussed.

## Connection Between Performance Parameters and Morphology of the Active Layer

PCE of photovoltaic devices is calculated as the ratio between the maximum power *P*_max_ generated by a solar cell and the incident power *P*_in_ Eq. 1. Alternatively, the PCE is determined from the Current density–Voltage (*j*–*V*) curves attending to the measured short-circuit current density (*J*_sc_), open circuit voltage (*V*_oc_), and Fill Factor (FF). From this equation, it is clear that all parameters *V*_oc_, *j*_sc_, and *FF* may limit the device performance as it will be discussed next.

1 $${\text{PCE}} = \frac{{P_{\hbox{max} } }}{{P_{\text{in}} }} = \frac{{V_{\text{oc}} j_{\text{sc}} FF}}{{P_{\text{in}} }}.$$

The first physical process that will limit the photocurrent is the optical properties of the active layer as it will ultimately determine the maximum photons that can be absorbed by the material. Without any doubt, the absorption is determined by the bulk properties of the active layer. For example, the optical density can be increased by increasing the thickness of the active layer or by creating an optical cavity [17, 18]. On the other hand, photobleaching is a commonly observed factor during degradation tests that gives rise to serious reduction in photocurrent. The relationship between optical properties of the active layer and photocurrent is probably the most intuitive and easy parameter to explain. However, from this point and due to the very complex nature of the donor and acceptor blend, efficiency will often be determined by a balance of different electrical processes: transport, recombination, and extraction of carriers.

Bulk morphology of the organic layer is undoubtedly one of the aspects most studied in OPV due to the high impact on device performance [19, 20]. Mixed donor/acceptor domains are required to provide optimum surface area to enable efficient charge separation [21]. On the other hand, continuous phases are required to allow charge transport toward the electrodes [22]. The combination of both factors will determine the final photocurrent of the device. Indeed, any reduction on the donor/acceptor surface area will proportionally decrease the photocurrent as it will decrease the photogeneration rate, for example, formation of fullerene aggregates in polymer:fullerene blends has been shown to consistently lead to a reduction in the observed *J*_sc_ by increased geminate recombination [23–25]. On the other hand, enhanced crystallinity of the polymer phase has been correlated with high mobility [26] and improved device performance [27], leading to reduced charge recombination processes [28].

**Fig. 1:**
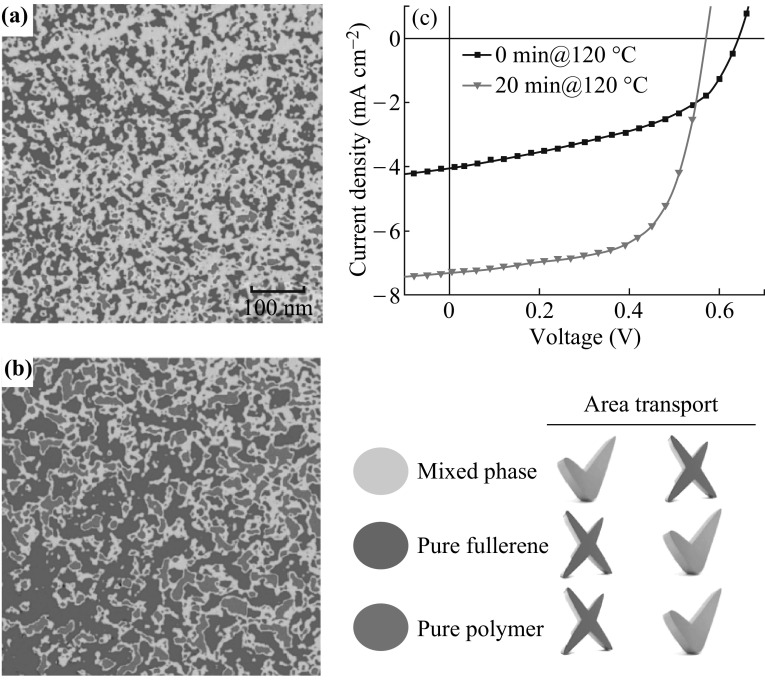
Morpohology visualization using high-resolution spectroscopic imaging with analytical transmission electron microscope for a sample P3HT:PCBM: **a** as casted, and **b** thermally annealed. **c** *j*–*V* curves for devices fabricated with same conditions as **a**, **b**. Adapted with permission from Ref. [29]

Applying high-resolution spectroscopic imaging using analytical transmission electron microscope, Pfannmöller et al. [29] have revealed morphology information which helps to understand charge separation and transport. Figure 1 shows images of two P3HT:PCBM samples processed differently, in which one is as casted and a second has been thermally annealed. This advanced imaging technique is able to distinguish between enriched polymer (green) or enriched fullerene (red) domains and those composed with a more amorphous distribution (yellow), i.e., intermixing of P3HT and PCBM. High photocurrent would be expected from the as-casted device as it shows a significant contribution of highly intermixed donor and acceptor and high interfacial area to provide enhanced charge separation. However, transport properties are hindered due to the highly amorphous nature of the phases, making difficult an efficient transport of carriers toward the electrodes. On the other hand, the large size of P3HT-rich and PCBM-rich domains for the annealed device provides smaller interfacial areas but the transport properties are favored since charge transport can be facilitated in the larger but purer material domains. As a consequence, the obtained photocurrent and FF are higher for the annealed device (Fig. 1c) as compared with as casted.

**Fig. 2:**
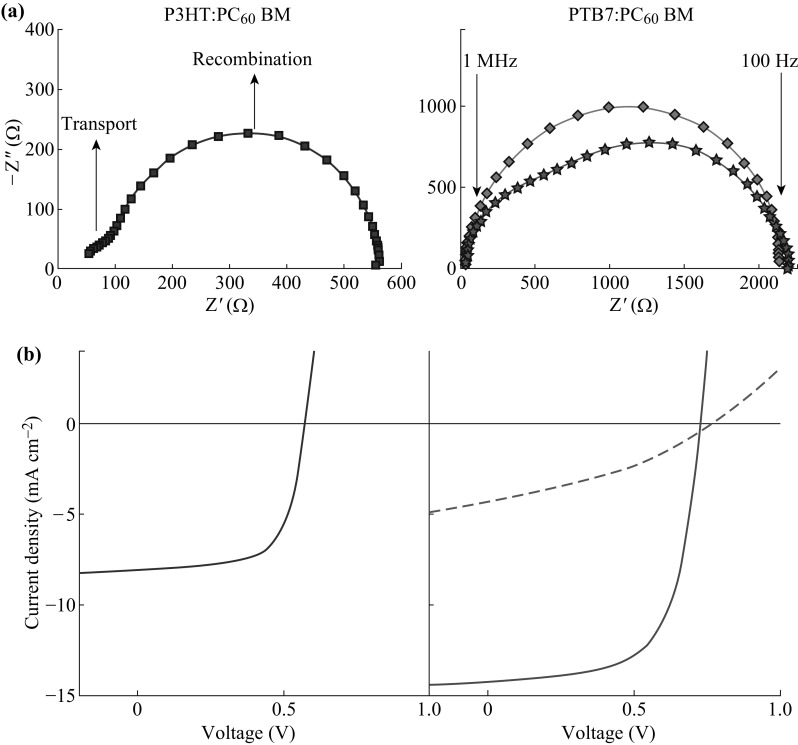
Representative P3HT:PC60BM, PTB7:PC60BM devices that illustrate the differences in the observed IS response for devices measured at 1 sun light intensity, at applied voltages close to *V*_oc_. Figure adapted with permission of Ref. [35]

Transport properties are best probed using electrical techniques which are able to monitor quantitatively resistive processes. Transport issues will generally manifest as an increase in the series resistance of the device and can ultimately affect both photocurrent and FF. Impedance spectroscopy (IS) has previously proved very useful for other photovoltaic techniques to discern between different resistive processes provided they take place at different characteristic times/frequencies [30, 31]. IS can provide simultaneous information on charge storage [32], carrier lifetimes [33], recombination kinetics, and resistive processes limiting the device performance in an operating solar cell [34]. In this technique, a DC bias is applied to a working device, either in the dark or light, to probe different positions in the *j*–*V* curve. At this DC bias, a small AC perturbation is applied (i.e., 20 mV) covering a wide range of frequencies (MHz to mHz) and the differential current output is measured. The technique provides well resolved arcs in the Nyquist plot (*Z*′ vs –*Z*″) for electronic/ionic processes occurring at different characteristic times (or frequencies). Figure 2 shows representative examples where issues related with transport of carriers are manifested as an arc in the high-frequency region of the Nyquist plot [35]. In these plots, the *x*-axis is related to resistive processes and the *y*-axis with the ability of the system to store carriers (capacitive response). Therefore, the larger the size of the arc, the higher the resistance associated to the process. High transport resistance is observed for non-optimized devices as that shown by the red trace and this correlates with an increased series resistance and reduced photocurrent and FF. A fully optimized device as that of the PTB7:PC_60_BM purple trace will only show one arc that corresponds to the recombination resistance (*R*_rec_) as it will be discussed below. The improvement of transport properties is generally attributed to an adequate modification of the polymer packing in the blend [36, 37].

Once light has been absorbed, carriers separate and transport toward the electrodes. Recombination events can take place and these will determine the collected carriers affecting all *J*_sc_, FF, and *V*_oc_. The fact that both recombination current and photocurrent are present under illumination does permit the differentiation between both during *j*–*V* curves analysis. However, using impedance spectroscopy, these can be separated as the method experimentally determines differential quantities and the photocurrent is constant at a given DC voltage. Hence, only the voltage-dependent term is measured. Thus, only recombination current (*j*_rec_) is measured and the recombination resistance (*R*_rec_) is defined from the derivative [38],

2 $$R_{\text{rec}} = L\left( {\frac{{{\text{d}}j_{\text{rec}} }}{{{\text{d}}V_{F} }}} \right)^{ - 1},$$

where *V*_F_ is the applied voltage corrected to take into account the series resistance of the device [39]. As *R*_rec_ is the derivative of the recombination flux, *R*_rec_ represents the opposition of the system to recombine carriers and high recombination resistances are desired for correct device operation. As *R*_rec_ is related with the recombination current at a given DC bias, this parameter will provide direct information on the recombination of carriers determining *V*_oc_, *FF* and *j*_sc_. Note that *j*_sc_ will be determined by recombination of carriers when the series resistance is very high. For OPV, it has been shown that close to *V*_oc_ bimolecular recombination is the main recombination pathway [40].

**Fig. 3:**
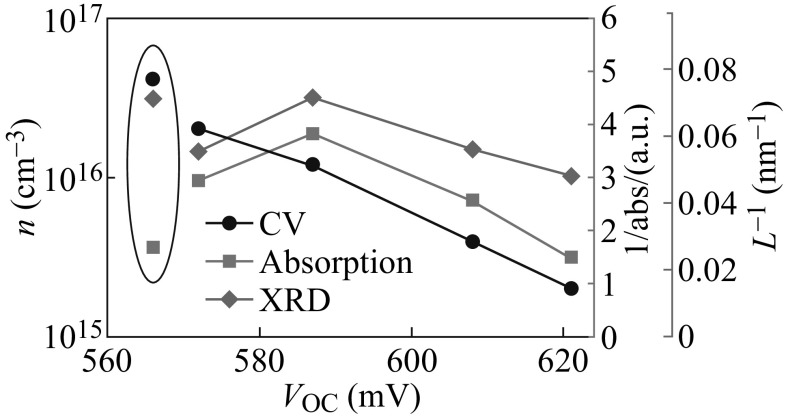
Relationship between domain size, as calculated by absorption, XRD, defect densities, *V*_oc_ of the devices for devices containing the system P3HT:PC_60_BM. High defect states give rise to higher recombination processes reducing the *V*_oc_. Reproduced with permission from Ref. [28]

Finally, it has been shown that *V*_oc_ is determined by the splitting of the Fermi levels of electrons and holes at the contacts and recombination can limit the *V*_oc_. Regarding the relationship between crystallinity and *V*_oc_, for semicrystalline blends like P3HT:PCBM the domain size is related with the number of defect states at the bulk: large domains as calculated by XRD or absorption measurements show decreased defect densities (Fig. 3) and recombination processes are inhibited. Indeed, high defect densities enhance recombination processes leading to reduced *V*_oc_ [28].

## Effect of Morphology in the Nano- and Microscale

**Fig. 4:**
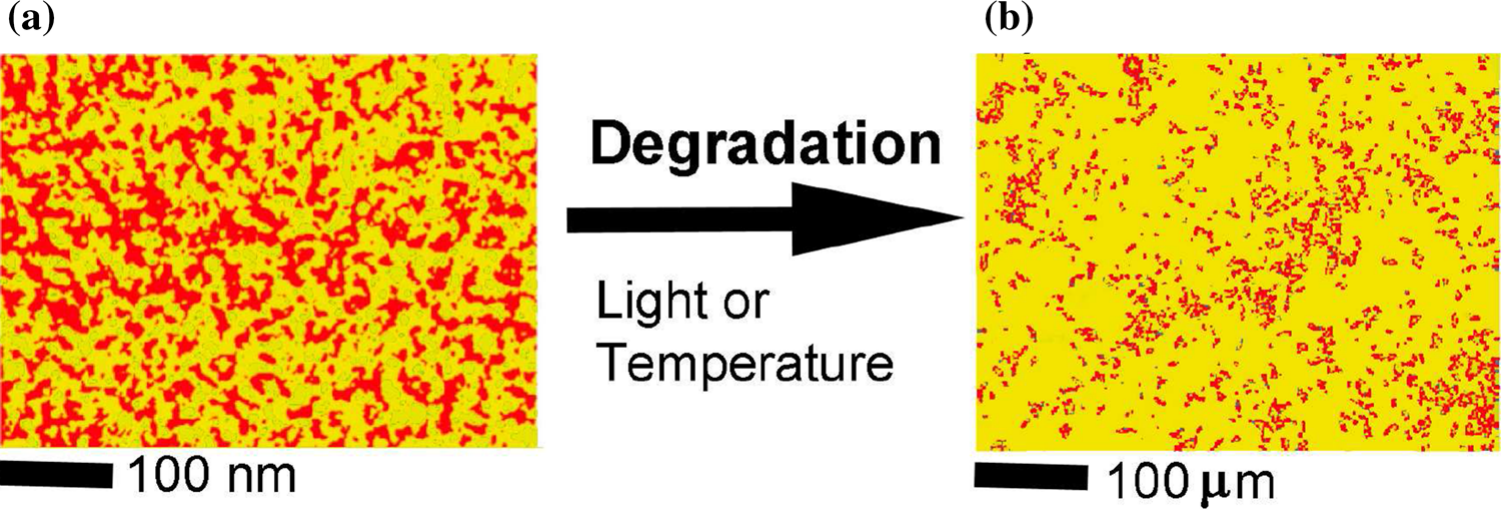
Diagrams representing adequate morphology for device operation in the nanoscale (**a**), microscale phase segregated phases (**b**) produced by degradation external agents

As discussed above, the effect of the morphology between the donor and acceptor is key for adequate device operation. In particular, the short exciton diffusion length in organic semiconductors, in the order of tens of nanometers, defines the maximum domain size to avoid recombination processes [41, 42]. Different techniques such as the use of thermal treatments or use of additives have proved very useful at providing the correct morphology (Fig. 4a). Unfortunately, the beneficial morphology for device performance is usually a kinetically frozen state in which thermodynamic equilibrium has not been reached. Indeed, thermodynamically pure domains are more stable than intermixed phases, and changes in morphology leading to migration of donor and acceptor molecules will be one of the main causes of degradation. As it will be discussed below, external factors such as light or temperature will lead to totally phase segregated phases where large microcrystallites of fullerene in the microscale are observed, red domains in Fig. 4b. Generation of these microscale domains will negatively impact charge separation as the contact area of donor and acceptor molecules will largely reduce the photocurrent. In addition, morphology as that shown in Fig. 4b will lack from pathways for carriers to reach the external contacts being the transport properties seriously damaged. Indeed, the red domains are not interconnected, the transport will be impeded and the most free carriers will inevitably recombine leading to very low performance parameters.

## Morphological Stability

As discussed above, the electronic properties of organic photovoltaics are strongly influenced by various physical processes and one of the general aims in the field has been to correlate structure and function. Solar panels under operation conditions usually reach temperatures as high as 65–85 °C and thermal degradation is a key factor that needs to be controlled [43]. Studies on highly efficient OPV devices show severe efficiency losses after a short operation time, being a morphological reorganization which is the main factor reducing the device efficiency [44]. Different techniques have been developed to attempt to lock the nano-morphology and avoid generation of microscale domains under thermal stress as it will be discussed below. For example, high glass transition temperature polymers have been used with the aim to avoid fullerene diffusion at temperatures close to the glass transition temperature (*T*_g_) of polymer [25]. It is important to note here that it is difficult to predict the thermal behavior of a bulk-heterojunction (BHJ) solar cell through the bare analysis of the *T*_g_ of pristine materials due to several factors. For example, it is difficult to measure the *T*_g_ of an amorphous polymer.

**Fig. 5:**
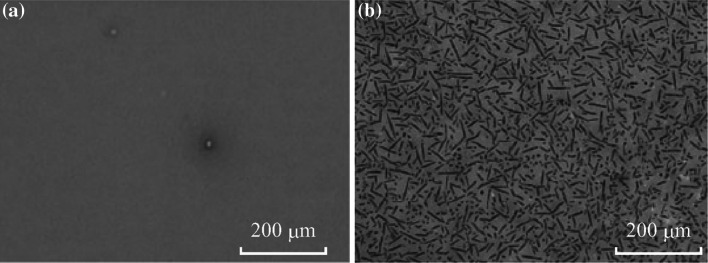
Comparison of optical images of films after annealing at 130 °C for 80 h of **a** fullerene-attached diblock polymer, and **b** P3HT:PCBM. Adapted image reproduced with permission of Ref. [51]

Several techniques have been developed over the last decades to provide the adequate morphology of polymer:fullerene blends including the use of thermal treatments or use of additives. Regarding the stability of this beneficial morphology, it is believed that the tendency of fullerenes to diffuse through the polymer phase resulting in a formation of large fullerene crystals via Ostwald ripening is regarded as the primary cause of morphology modification. In Fig. 5, optical images of two films are shown to behave totally different toward thermal stress. A specially designed blend containing a fullerene attached to a diblock polymer (Fig. 5a) does not show formation of large microscale PCBM aggregates after a treatment at 140 °C during 80 h. Alternatively, a P3HT:PCBM blend shows generation of large aggregates (Fig. 5b) reducing all performance parameters. In order to avoid this fullerene diffusion, several routes have been followed to lock the morphology with different degrees of success. These pathway include the use of ternary blends with compatibilizers such as block copolymers [45], amorphous fullerene derivatives [46], donor–acceptor systems with enhanced supramolecular interactions [47], functionalized side chains on the polymer [48], or thermocleavable polymers [49]. We will next discuss some parameters affecting the blend morphology that has not been explored much in detail and requires further attention. We will discuss a new technique to provide information on thermal stability in general.

Regarding the morphological stability of different type of OPV devices by far the most studied systems regarding morphology stability by solution process are devices based on polymer donor and fullerene acceptor materials. However, it is important to highlight that efficiency of OPV containing non-fullerene acceptors, small-molecule donor material and all polymer solar cells have sharply progressed very recently. Morphological stability is likely to follow the same rules as those described for polymer:fullerene devices. However, examples on thermal degradation studies on these systems are rather scarce in the literature [50]. To provide some guidelines on what expect for each system here we compare small molecule devices processed from high vacuum conditions with some solution-processed devices. Evaporated small molecules have shown superior thermal stability compared to polymer-based devices as they are actually processed at temperatures above 100 °C. In addition, these evaporated small molecules do not need long hydrocarbon side chains to confer solubility in solvents which typically would reduce the *T*_g_ in the polymer analogues. Alternatively, devices based on solution process small molecules suffer from the need of these solubilizing groups which reduces the crystallinity and *T*_g_ of the films. Therefore, it is expected that morphological stability to be lower for this last group and this may be the reason behind the lack of reported data for high efficiency devices.

### Additives Effect

**Fig. 6:**
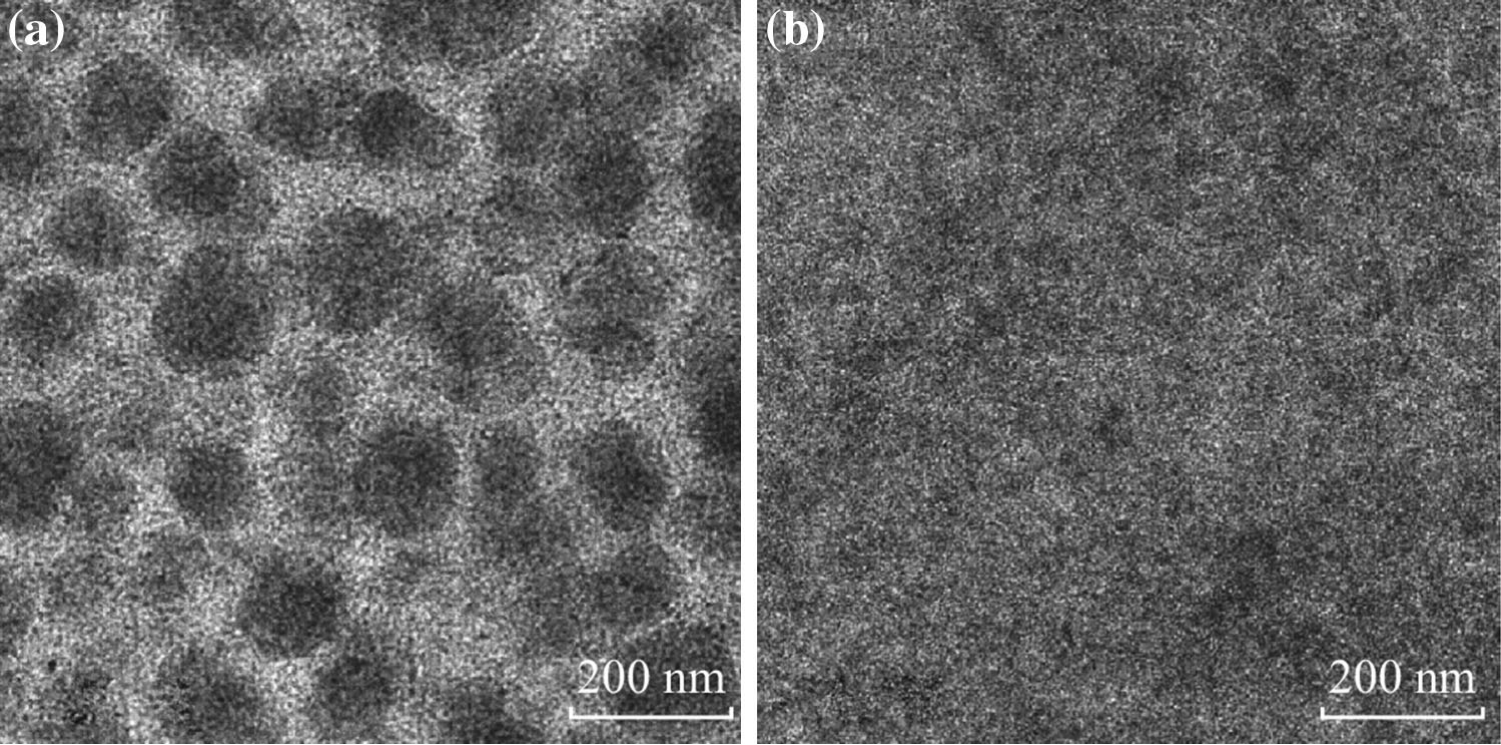
TEM images of PTB7/PC71BM blend film prepared from chlorobenzene without (*left*), with (*right*) diiodooctane. The *scale bar* is 200 nm. Reproduced with permission from [53]

The use of additives has long been known to improve the device performance [52] by retarding the crystallization kinetics of one of the two components of the active layer providing an adequate morphology in the nano- and microscale (Fig. 6). Additives with high boiling points are used to preferentially dissolve the PCBM providing a good intermixing and avoiding generation of large domains of pure acceptor molecules [53]. Commonly used high boiling point solvents such as dichlorobenzene can also be regarded as additives [54]. A recent review has been published on the use of additives with several high efficiency systems [55].

Importantly, small amounts of additive usually remain in the film and the effect on the device stability has not been much studied. In a recent study it has been shown that residual 1,8-diiodooctane (DIO) remains after vacuum electrode deposition and the presence of this additive shows a very negative impact on device stability [56]. Indeed, devices containing DIO additive show improved initial device performance of 7.7 % as compared to devices not containing the additive 3.4 %. However, degradation decay using the protocol ISOS-D-1 (shelf life in air conditions) provided devices with similar efficiencies after 300 h, 3.5 versus 2.8 %. The authors claim that diffusion of O_2_ and H_2_O takes place differently depending on whether additive has been used or not. However, intrinsic morphology of degraded devices was not studied in detail.

Alternatively, residual DIO additive has been successfully removed by using an orthogonal solvent which is able to remove the additive not affecting the organic layer. This methodology seems to be general as it has been reported for 5 different polymers and morphological stability is enhanced as well as the efficiency and reproducibility after spin-coating of inert solvents [57]. The orthogonal solvents must follow the following requirements: 1-Must not dissolve de organic layer, 2-Must be fully miscible with the additive, and 3-Swelling of the organic layer should be avoided as this would modify the morphology. In this regard different alcoholic solvents have been studied for the benzodithiophenes (BDT)-based polymers and the use of methanol seems to be the best choice regarding the photovoltaic performance compared to isopropanol [58].

### Use of Crosslinkers

**Fig. 7:**
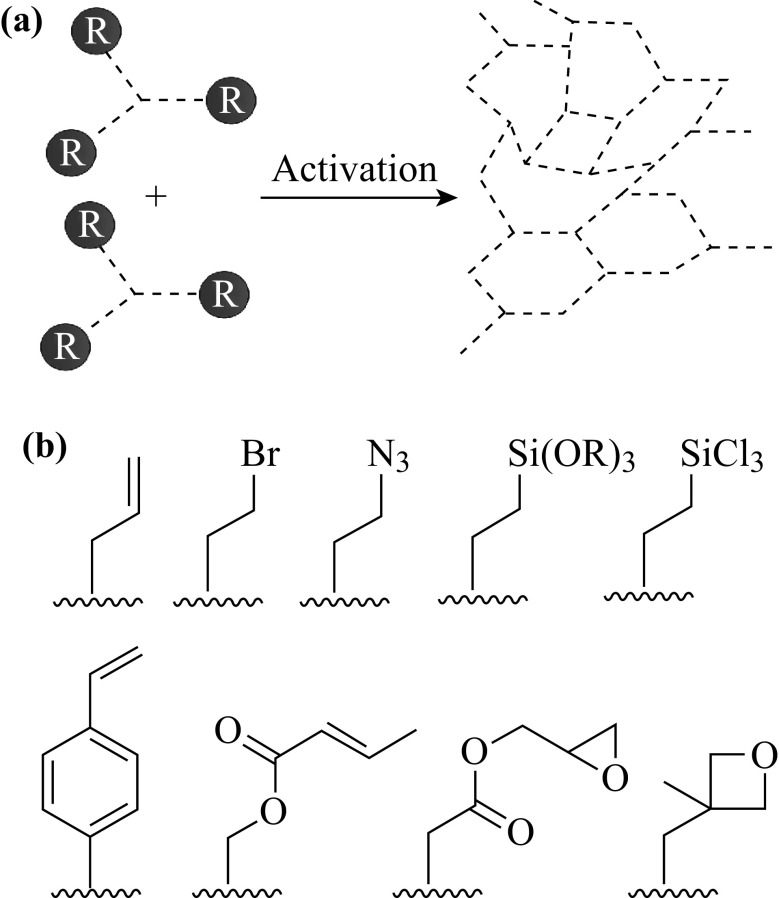
**a** Sketch representing the crosslinking process that provides reticulated networks. **b** Reactive functional units commonly used as crosslinkers

Crosslinkers are polyfunctional materials containing reactive structural units that provide permanent chemical bonds in the blend, see the sketch in Fig. 7a. The functional groups are usually not selective and can react with all donor, acceptor and/or other crosslinking units. A reticulated network is generated which avoids diffusion of molecules locking the morphology avoiding generation of crystallites in the order of several microns. As discussed above for Fig. 5 the use of block copolymers containing crosslinking units can avoid morphology evolution under thermal stress [51]. If no special precautions are taken, fullerene aggregates are visible being a clear sign that the initial nanoscopic morphology beneficial for device performance is no longer present. A recent comprehensive review on the use of crosslinkers applied to OPV have been published by Wantz et al. [59] Several reactive groups such as those depicted in Fig. 7b can be used including alkenyl, bromide, azide, silanes, styrenes, acrylates, epoxy, oxetanes, and many others. The physical process that promotes activation will depend on the reactive group used and processes include thermal, UV light, photoinitiators light, acid or base initiators.

Latest examples include the work by Durrant et al. where illumination with UV light during film processing is shown to improve device stability due to oligomerization of PCBM molecules [60]. Stability test were carried out during 20 h only and during this time efficiencies compared to the control were already very similar, 4.8 and 4.4 %, respectively. This result is not really surprising as oligomerization of PCBM promoted by light is probably a reversible reaction following Diels–Alder chemistry. Alternatively, specially designed fullerenes with crosslinking units can be introduced to lock the morphology conformation of the bulk of the active layer [61] or the interface with the electron selective layer [62]. In addition, the crosslinking unit may be added in the donor material [48]. Finally, unselective and highly reactive units such as azide crosslinkers can be used to create chemical bonds with several chemical functionalities [63].

The use of crosslinkers to lock the morphology may be regarded as a final solution. However, this approach usually shows some practical limitations which are often difficult to overcome. For example, after the expected crosslinking chemical reaction some unreacted units may still be present in the active layer which could be negative for the device performance. Indeed, introduction of molecules which do not participate in the charge generation and transport usually show a negative impact on the initial device performance. See for example the high efficiency system described by McCulloch et al. in which it is observed an efficiency drop from 7.0 to 5.7 % during the curing process [63]. Also important is that the final device performance after thermally aging at 85 °C during 130 h using the azide crosslinker cannot be considered as high efficiency any longer (4.1 %). Therefore, it appears that more work in this direction is needed to provide a definite solution.

### Thermal Stability Probed by Capacitance–Temperature

**Fig. 8:**
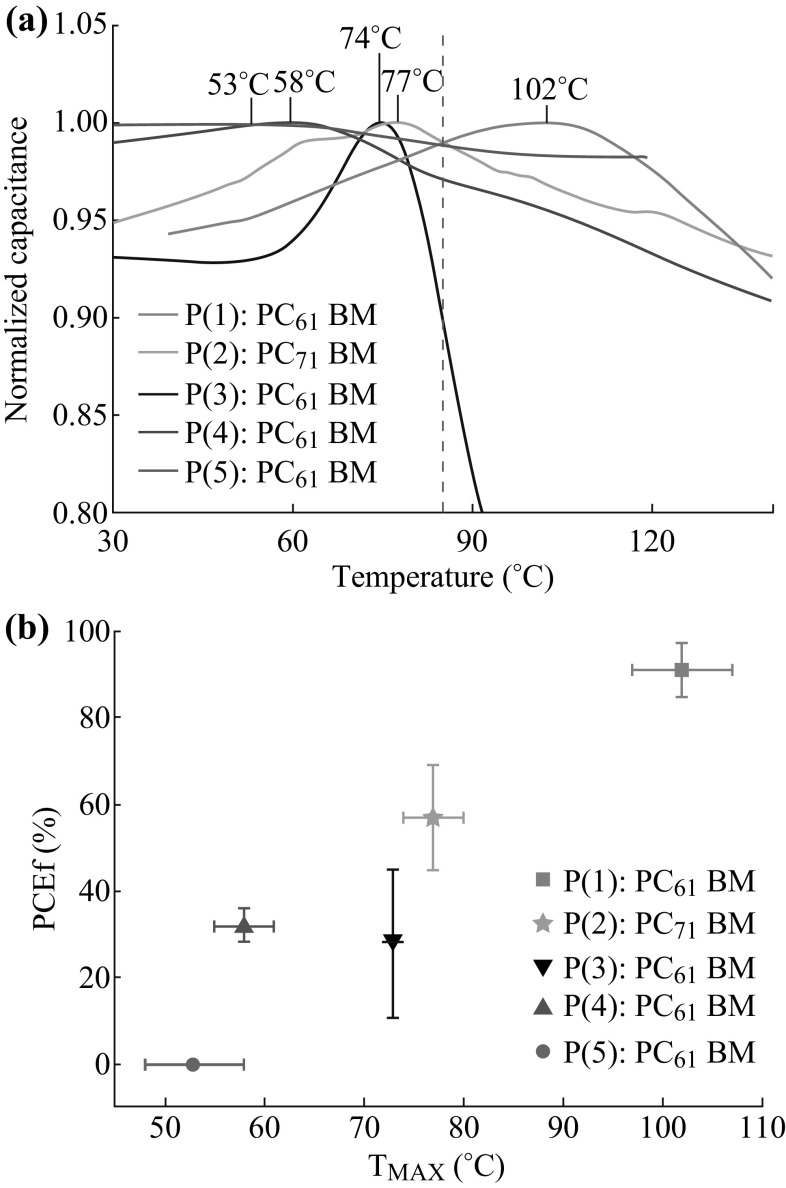
Comparison of devices fabricated using different donor:acceptor systems in terms of their response to **a** capacitance–temperature measurements and **b** efficiency decay as the temperature at which the capacitance is maximum (*T*_MAX_). With permission from Ref. [64]

In order to have a good description of the thermal stability of the complete device a new technique has been developed recently which enables the study of the integrity of whole stack as a function of the temperature [64]. The method probes the geometrical capacitance (*C*_g_) of the active layer to monitor morphological changes in operating devices. The geometrical capacitance is sensitive to modification in the dielectric constant and thickness variations of the active layer, probing nano- and microscale morphology domain. Indirectly, *C*_g_ is also sensitive to modification at the contacts as charge injection properties may impede the correct measurement of *C*_g_. Figure 8 shows Capacitance–Temperature measurement for a set of five different donor:acceptor systems. The temperature at which the whole device becomes thermally unstable is defined as *T*_MAX_. The data are arbitrarily normalized to the value of *T*_MAX_ as the value itself does not seem to be correlated to degradation kinetics. The efficiency decay data were fitted to an exponential decay to provide kinetic information. It was found a direct correlation between *T*_MAX_ and the kinetics decay and final efficiency value (PCE_f_) obtained during constant thermal stress at 85 °C. In summary, devices are thermally stable when the temperature of the thermal stress is below the *T*_MAX_. This capacitance method gives valuable information to predict the thermal stability of BHJ solar cells using an accelerated test.

## Recent Techniques to Probe the Contact/Active Layer Interface

The link between morphology modification and performance has usually been discussed in terms of the bulk properties of the active layer [65], but very little attention has been paid to the evolving properties of the active layer/outer contact interfaces in the nanoscale domain. For example, a recent study has shown that migration of polymer/fullerene molecules toward the electrodes during thermal aging correlates with a loss in *V*_oc_ for degraded devices [66].

**Fig. 9:**
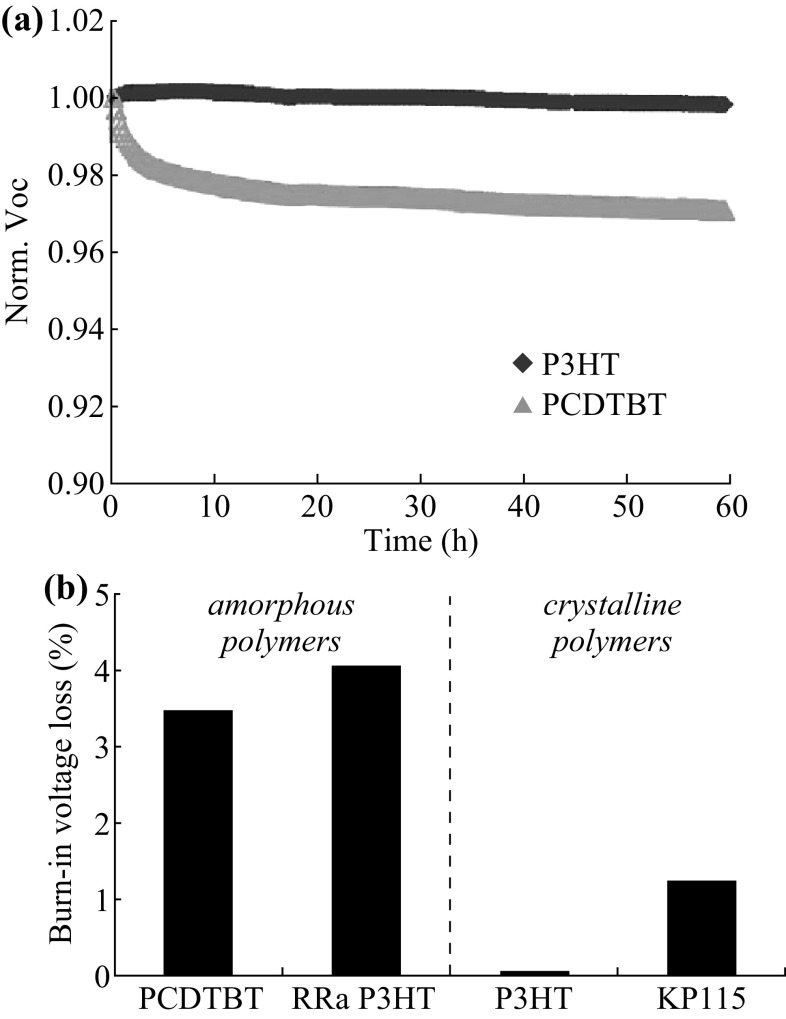
**a** Normalized open-circuit voltage losses over illumination time for PCDTBT and P3HT solar cells. **b** Data measured with a solar simulator before and after burn-in for amorphous and crystalline materials. Reproduced from Ref. [67] with permission from The Royal Society of Chemistry

Fast initial performance losses are present in many high efficiency materials and this efficiency decay is commonly denoted as burn-in. The actual mechanism in which this burn-in takes place has been very little studied. However, it appears to be related with all the different layers involved in the polymer solar cell and, specially, the bulk of the active layer. Indeed, it has been proposed to be caused by light-induced traps and its characteristics depend on which polymer is used [67]. Heumueller et al. showed some examples where there was a direct correlation between the presence of these light-induced traps and the open-circuit voltage loss in devices made with amorphous polymers (Fig. 9). Alternatively, solar cells made with crystalline polymers do not show characteristic open-circuit voltage losses pointing to a superior stability for crystalline materials.

In spite of this recent work degradation studies to specifically understand burn-in of the device performance are very scarce in the literature and further work is needed in this direction. For example, it is of utmost importance to understand if these “light-induced” traps are somehow related to the charge transfer complexes described above or if the superior stability is related with a better morphology stability, i.e., the different materials study may give different vertical segregation profile depending on the nature of the polymer.

Indeed, charge extraction can be modified during the lifetime of the device by a migration of either polymer [66] or fullerene [68] adhering to the top contact to generate a skin layer. The overall effect is the formation of barriers for selective extraction of carriers or hindered transport regions depending on the device architecture. In addition, selectivity of the contact may be lost increasing the leakage current which can ultimately limit the achievable photocurrent [35, 69, 70].

Unfortunately, direct observation of the active layer/contact interface is not trivial and latest developments have been focused to obtain physical techniques to offer information at the interfacial level. Hence, one of the issues that still require intensive work is the development of physical tools to probe the specific mechanisms behind active layer intrinsic evolution and its relationship to device performance [60]. In this section, we will provide a range of new techniques which certainly need to be used in the future to further understand the effect of the external interfaces.

### Scanning Transmission Electron Microscopy Spectral Imaging

**Fig. 10:**
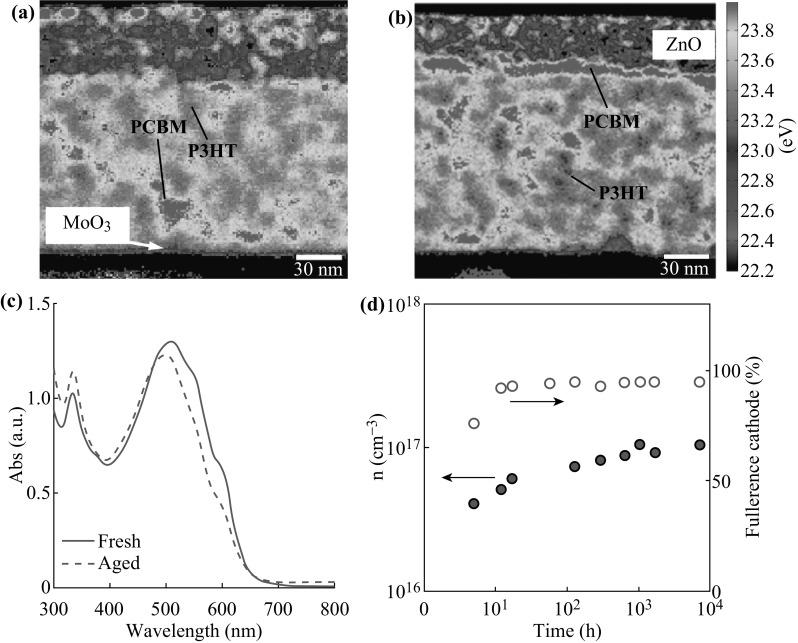
Maps showing plasmon peak positions of ultrathin cross sections of devices P3HT:PCBM devices: **a** fresh, and **b** aged. **c** Absorption spectra of fresh, aged films cast from CHCl_3_. **d** Evolution of defect density values (*n*), calculated fullerene content at the cathode interface extracted from capacitance–voltage measurements carried out in the dark of devices cast from CHCl_3_. Reproduced with permission from Ref. [12]

Applying high-resolution spectroscopic imaging using analytical transmission electron microscope Pfannmöller et al. [29] have revealed morphology information in the nanoscale morphology domain which helps to understand charge separation and transport (Fig. 1). This advanced imaging technique couples spatially resolved electron energy-loss spectroscopy with the analytical scanning transmission electron microscopy [12]. This combination is termed STEM spectral imaging (STEMSI) and offers spatially resolved low-energy loss spectroscopy at the nanoscale. Figure 10a, b show the cross section corresponding to two devices analyzed using STEMSI for a shelf life degradation study as it will be discussed below. In these images it is possible to distinguish between enriched polymer (green) or enriched fullerene (red) domains with those composed of a more amorphous distribution (yellow), i.e., intermixing of P3HT and PCBM. This technique provides a first qualitative visual indication on whether the blend morphology is as correct as expected. Indeed, large P3HT-rich and PCBM-rich domains are desired in terms of transport properties and large intermixed domains for efficient charge separation. Obviously, the technique is very powerful but the main disadvantage is the destructive nature of the measurement which does not allow monitoring the “same” device during device degradation experiments. Similarly, by preparation of rod-shaped specimens the same group has provided 3D structural correlations and quantitative compositional mapping at a resolution of approximately 7 nm using tandem cells [71].

The effect of the incorrect vertical segregation profile has been studied by STEMSI in shelf life degradation studies and evolution of the morphology in the bulk and the effect of vertical segregation profile have been separated. The experiment was carried out in conditions to probe shelf life degradation of OPV by excluding extrinsic factors (i.e., light, heat, water and oxygen). Of particular relevance to study intrinsic stability is a variation of the protocol ISOS-D-1 in which devices are not stressed keeping them in the dark under nitrogen atmosphere and ambient temperature [43]. A study completed using chloroform as solvent provides a kinetically frozen morphology even after a thermal treatment. Storage of the device in the dark and in the glovebox enables a morphology rearrangement which doubles the efficiency over a period of 1 year [12]. As can be observed in Fig. 10a evolution of morphology in aged samples shows that demixing and distribution of domains does not change in agreement with absorption measurements Fig. 10c. On the other hand, a PCBM-rich layer is developed at the ZnO contact (Fig. 10b) in agreement with fullerene cathode coverage calculated by *C*–*V* measurements, a technique which will be discussed below. Interestingly, improvement in device efficiency is observed due to a modification of the selectivity of the cathode contact. In particular, *j*_sc_ nearly doubles by a reduction of the leakage current and decreasing the recombination of carriers. Importantly, by carrying out impedance spectroscopy measurements if an incorrect vertical segregation profile is observed a feature in the low-frequency region is observed which can be attributed to the effect of an undesirable contact resistance.

### Impedance Spectroscopy

**Fig. 11:**
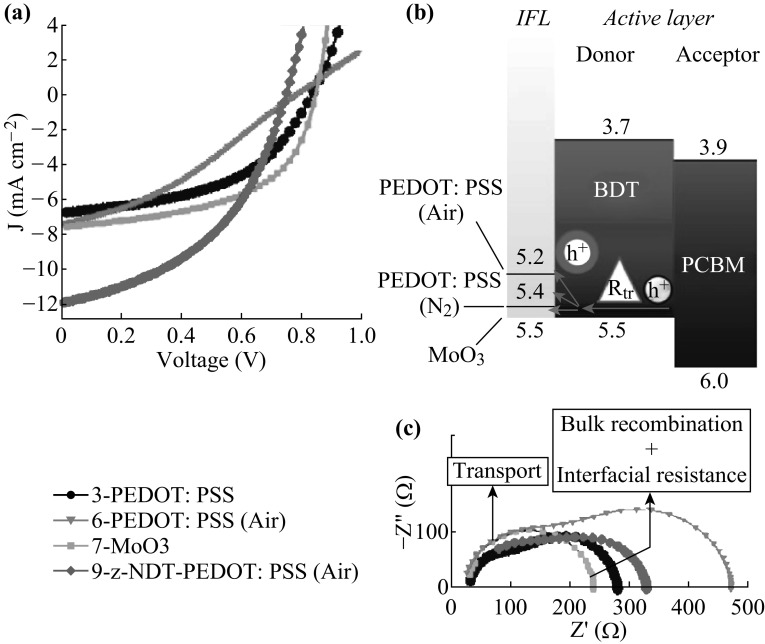
**a** *j*–*V* response of small-molecule donor OPVs to understand the effect of active layer thickness. **b** Impedance spectra of devices shown in (**a**) measured at 1 sun illumination at *V*_oc_ conditions. Reproduced from Ref. [31] with permission from The Royal Society of Chemistry

As discussed above, IS is a very useful technique to understand effects related to bulk property of the active layer proving both nanoscale and micromorphology domain. Alternatively, the effect of poorly extracting contacts can also be observed using this technique which helps the identification of additional resistances associated to the contacts. Using impedance spectroscopy, it is demonstrated that the low fill factor (FF) typically observed for the BDT family in small-molecule solar cells is partially due to hindered charge transfer through the anode interfacial layer (Fig. 11) [72]. By carefully tuning the anode in BDT(TDPP)_2_ solar cells, the FF can be increased from 33 to 55 %. Interestingly, the modification of the hole selective layer shown in Fig. 11 leads to a reduction of the resistance in the low-frequency region, which distinctly affects the FF of the device. Likewise, for the P3HT:PCBM system described in Fig. 2, an additional resistance is observed in the low-frequency region if an incorrect vertical segregation profile is observed by imaging techniques [12]. Therefore, IS has been described recently as a useful tool to understand vertical segregation profiles l.

### Capacitance–Voltage Analysis

**Fig. 12:**
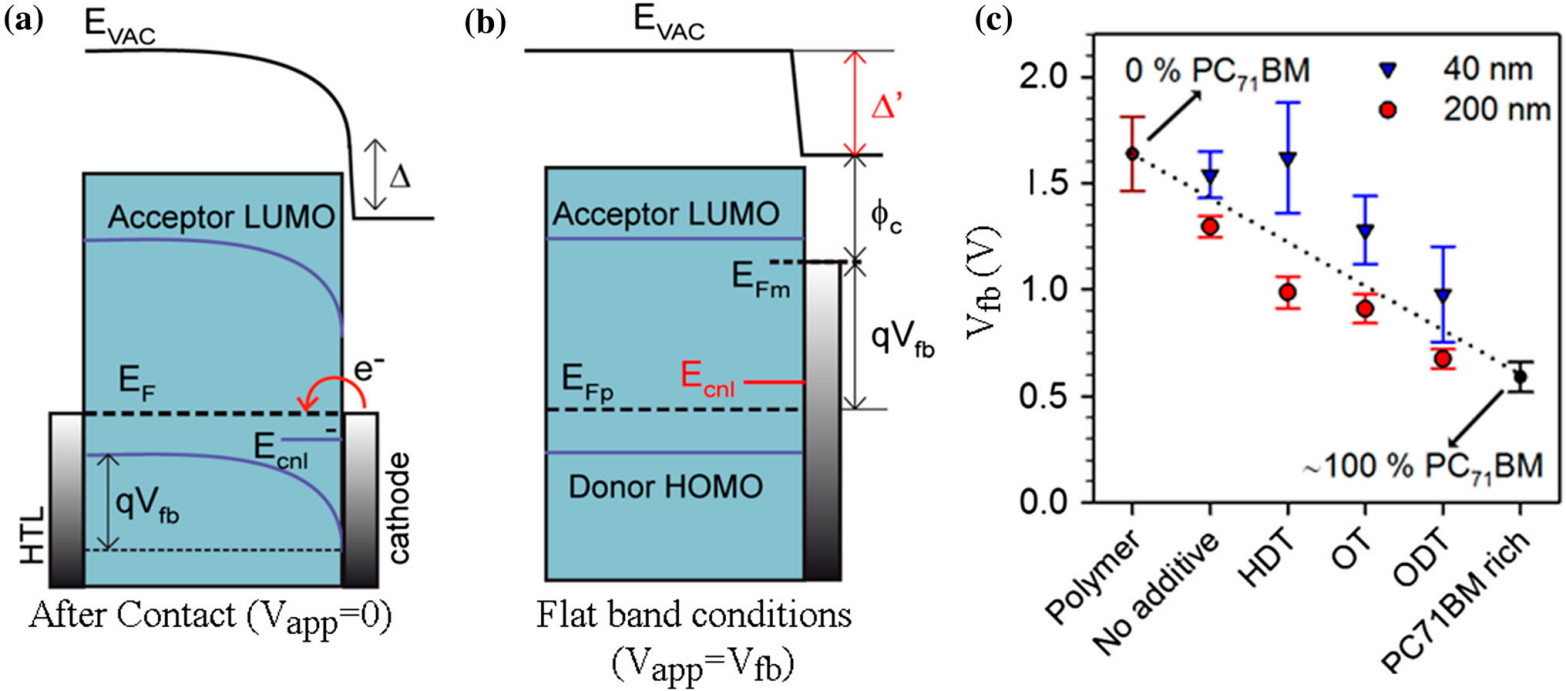
Energy level diagrams representing the cathode equilibration of a bulk-heterojunction solar cell: **a** before contact equilibration, **b** after contact deposition. **c** *V*_fb_ results extracted from *C*–*V* measurements in the dark for devices processed under different conditions. Two sets of devices with different active layer thickness are included that show that the method is valid for different active layer thicknesses. Adapted from Ref. [70]

Energy-level equilibration at the active layer/cathode takes place by generation of a dipole and band bending as shown in Fig. 12 and takes place in the nanometer scale domain [73]. The magnitude of the dipole and the band bending contribution will greatly depend on the material which is physically present at the interface. For example, cathodes covered with high proportion of polymer show large dipoles and low band bending contribution. In this respect, capacitance–voltage (*C*–*V*) analysis allows determining the fullerene content at the cathode which is directly related to the contact selectivity and leakage current [70, 73]. In this technique, a small AC voltage perturbation is applied to a working device at varying DC voltage biases and the differential current output is measured under dark conditions. As revealed previously using a frequency between 100 and 1000 Hz for OPVs, the capacitance related to the depletion region at the cathode contact is probed. Indeed, Mott–Schottky analysis can be carried out to measure the required potential to provide flat bands (flat band potential, *V*_fb_) and calculate the dipole. In the experimental method, two reference devices are used to calculate the *V*_fb_ for the extreme coverage cases, i.e., pure polymer and a device containing high proportion of fullerene (donor:acceptor ratio 1:6). Then, measurement of *V*_fb_ of blends allows the interpolation into the maximum content limits to calculate the degree of polymer/fullerene coverage. Hence, the technique offers information on the energy-level equilibration at the organic layer/cathode interface where a dipole layer is generated by reduction of the fullerene molecules present at that interface [73]. It was shown that devices containing large proportion of polymer molecules present at the interface with the cathode suffered from high leakage current by a reduced selectivity of the contact.

### External Quantum Efficiency

**Fig. 13:**
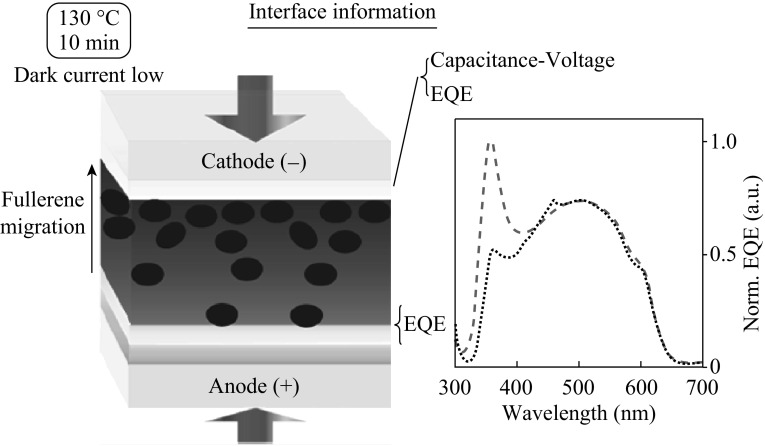
Sketch representing the method to monitor the presence of donor and acceptor molecules in close contact with a semitransparent electrode

Recently, a new technique has been developed that uses widely available equipment to measure EQE. The principle is to prepare semitransparent devices and measure EQE from the semitransparent top contact [74]. As most light is reflected by the metallic contact, only a small fraction of light reaches the active layer, and due to the high extinction coefficient of the materials, the measurement provides fundamental information of molecules in close proximity to the semitransparent contact (Fig. 13). Then the technique probes the nanoscale domain at the proximity of the contacts. A good agreement with coverage values calculated from *C*–*V* measurements has been observed. Interestingly, in this case, the observation of one contact is not limited to the active layer/cathode interface and can also be used for the anode/active layer interface. Alternatively, one of the limitations of the technique is that maximum absorption bands of donor and acceptor needs to be complementary. This recent work highlights that the vertical segregation profile depends on both processing conditions of the layer and substrate used to grow the different layers and has been studied in both regular and inverted configuration.

### Further Techniques to Test Contact Evolution

Several comprehensive reviews of advance physical techniques have been published in the field of OPV to understand morphology configurations [75]. Emerging technologies such as helium ion microscopy provides high sensitivity and low beam-induced damage of the samples but equipment is not widely available [76]. Tip-enhanced Raman spectroscopy (TERS) is another promising technique for nanoscale chemical analysis of surfaces. TERS exploits the effect that Raman signals of probed molecules can be enhanced by many orders of magnitude [77]. Very high resolution can be obtained since it only depends on the apex size and shape. By reduction of the diameter, chemical information with resolution in the range of 10 nm is achievable. This creates possibilities for various potential applications in biology and materials science fields, which are summarized in a recent review article [78]. Applicability of TERS to polymer blends and BHJs was demonstrated. Yeo et al. reported a 20 nm resolution for compositional profiling of a polyisoprene and polystyrene blend [79]. For a P3HT:PCBM blend chemical contrast at 10 nm resolution was achieved by Wang et al. [80]. In that work, P3HT-rich, PCBM-rich, and mixed regions as identified by Raman signals were compared with photoluminescence and topographic mapping of the layer surface.

## Conclusions and Further Work Needed

The present manuscript discusses some of the advances witnessed over the last 5 years to better understand morphological degradation of OPVs. A range of new techniques have been developed to separate effects related to morphology changes in the bulk of the active layer to those related to the interfaces with the external contacts. Overall, all factors contributing to an increased morphology stability at all levels (bulk and vertically) will help to increase the device stabilities. The use of polymers benefiting from a high transition temperature can definitely improve the thermal stability in terms of bulk morphology. Importantly, more work is urgently needed to stabilize the contact interfaces, i.e., the use of crosslinking molecules or SAM at the contacts may help to increase the stability toward modification of the leakage currents during device operation. Importantly, it is yet to be found that crosslinkers not only improve the morphological stability but also allow the achievement of high efficiencies. Efficiency of OPV containing non-fullerene acceptors, small-molecule donor material, and all polymer solar cells have sharply progressed very recently. Morphological stability is likely to follow the same rules as those described in the present manuscript, but further data are needed.
